# Thoughts on Ill-Health

**Published:** 1890-08-23

**Authors:** 


### Thoughts on Ill-Health.

### II.—AUDI ALTERAM PARTEM.

Those who try to realise the position of such of their fellow creatures as are never quite "fit" will, we feel sure, both recognise and pity the many difficulties and trials of that position. None the less, they may very possibly retort—"But is there not another side to the question, which you have left altogether out of account? Have the healthy nothing to bear from the sickly? Are not we often victimised by them, quite as much as they by us?"

Let us admit at once that there is some force in this rejoinder. Whilst it is always well for others to try and realise the hardships, the limitations, the disappointments of those who suffer from ill-health, nothing can be worse for those sufferers than to be always dwelling on their own troubles, and making their lives a burden to those around them as well as to themselves. There are some selfish invalids who manage to become the tyrants and torments of their respective households; and without dwelling longer upon such extreme cases, it is beyond dispute that the ill-health of many persons is made far more of a burden to their friends than it need be, by the way in which they take it.

The first enemy that ill-health brings in its train is perhaps Egotism, and a formidable enemy it will often prove. The man who never enjoys good health is forced to keep guard over himself, to ask whether this or that thing will hurt him, to watch whether this or that course of treatment seems to be the more hopeful. His thoughts being thus drawn towards himself, he becomes self-absorbed; *my* health, *my* interests, *my* concerns begin to take a larger place in the moving panorama of life than they do with a physically healthy man. The next thing is that they arrogate to themselves by far the larger part of his conversation; his ailments, his course of treatment, his inability to do this or that "on account of my unfortunately weak health," are the standing dish upon which you may reckon whenever you look in to have a chat with him. Even a little boy of twelve years old, whose doctor not very wisely told him to watch and report his symptoms, has been known to descant upon the half-dozen kinds of headaches from which he suffered, to a bored company of friends and relatives, until at last he was bluntly told to "eat his tea and be quiet." How often the unwilling auditors of a catalogue of complaints from some more venerable sufferer must have wished that they could put a stop to it in the same way! Egotism is always tiresome, but (let those in delicate health take this to heart) no egotism is so insufferable as that of the man who tells you, not of what he has done or seen, but of what he is feeling or has felt. Probably he cannot help taking a certain mournful pleasure in thinking over his symptoms; and as he is very often forced to think of them, no one, surely, would grudge him that pleasure. But he must not expect to communicate it to others. They are often really interested to hear how he is, and will be pleased or sorry, as the case may be, to learn that he is a little better, or not quite so well. But if he enters upon a deliberate and judicial examination of his case—"Well, thank you, I can hardly say that I am better. The acuteness of the headache has a little gone off—yes, I think I can certainly say that it is a trifle less violent to-day; but on the other hand, the weakness in the throat has certainly not diminished—I am not sure but what it has very slightly increased; and the last dose of medicine which I took during the night did not have the beneficial effect which I expected, although I fell into a fairly refreshing sleep about half-past ten, or perhaps twenty minutes to eleven, after taking half-a-cupful of Liebig, with a tea-spoonful of brandy in it,"—well, we are a little shy of putting the same question to him the next morning, unless we have several minutes and a little patience to spare. After all, there is far more to pity than to laugh at in his case; but for his own sake he should try to foster by all means in his power a sense of the relative proportion and the fitness for discussion of the various subjects which suggest themselves to his mind—a sense whose presence makes a delightful companion, whose absence, a bore.

To some, of course, this dwelling upon one's woes is no temptation. On the contrary, they dislike to have their ailments known and talked of, and will hardly admit, even to themselves, that they cannot do what others can. It is pathetic sometimes to see the desperate endeavours which they make to struggle against their difficulties; to watch the suffering and the weariness which so evidently overcomes them now and then, but which one dares not notice. The pity of their neighbours is bitter to them as wormwood; and yet they do unwisely, perhaps even wrongly, to reject it. The short sharp answer to an inquiry about their health, the irritable pushing aside of a suggestion that a certain bit of work is too hard for them, will soon shut off from them the compassion and sympathy that would make their lot an easier and a brighter one; and as for those who really love them, nothing is harder than to see a friend suffering, and yet suppress every sign of one's sympathy, for fear of making him wince the more. Stifled sympathy, crushed-down affection, is a pain quite as real as physical discomfort; and he who has to bear the latter should try not to make his friends suffer from the former.

